# The use of implementation science to close the research-to-treatment gap for cognitive impairment in psychosis

**DOI:** 10.1177/00048674231160987

**Published:** 2023-03-25

**Authors:** Isabel Zbukvic, Shayden Bryce, Joanna Moullin, Kelly Allott

**Affiliations:** 1 Orygen, Parkville, VIC, Australia; 2 Centre for Youth Mental Health, University of Melbourne, Melbourne, VIC, Australia; 3 enAble Institute, Curtin University, Perth, WA, Australia

**Keywords:** Pragmatic designs, cognitive remediation, clinical trials, cognition, schizophrenia

## Abstract

For people living with psychosis, cognitive impairment is common and can have significant impacts for functional recovery, impacting engagement with treatment and quality of life more broadly. There is now strong evidence for the effectiveness of cognition-focused treatments, such as cognitive remediation to improve clinical and functional outcomes for people with psychosis. However, engagement with treatment has been a long-standing issue in mental health care, including for people with psychosis, who often experience difficulties with motivation. While research on clinical effectiveness of cognition-focused treatment is growing, to date there has been little research focused on the implementation of such treatments and it is not clear how best to support uptake and engagement across diverse mental health settings. Implementation science is the study of methods and strategies to promote the adoption, application, and maintenance of evidence-based practices in routine care. To integrate cognition-focused treatments into routine practice, and improve engagement with treatment and the quality and effectiveness of care for people with psychosis, researchers need to embrace implementation science and research. This paper provides a succinct overview of the field of implementation science, current evidence for implementation of cognition-focused treatments for psychosis and practical guidance for using implementation science in clinical research. The future of psychosis research includes multidisciplinary teams of clinical researchers and implementation scientists, working together with providers and consumers to build the evidence that can improve the implementation of cognition-focused treatments.

Cognitive impairment is a core feature of psychotic disorders and often emerges before the onset of full-threshold disorder (Keefe and Fenton, 2007; Mollon et al., 2018; Reichenberg et al., 2010). While psychotic and other psychiatric symptoms respond to medication and psychotherapy, cognitive impairments do not and commonly interfere with functional recovery, including the ability to achieve vocational success and independent living (Allott et al., 2013). Best practice in the treatment of psychosis increasingly recommends addressing cognitive impairment (Galletly et al., 2016; Norman et al., 2017). Evidence-based treatments for improving cognition and associated functioning for people living with psychosis are available, including cognitive remediation and compensation, and social cognition training (Allott et al., 2020; Cella et al., 2020; Nijman et al., 2020; Vita et al., 2021). These treatments are associated with a range of positive outcomes, with meta-analyses showing improvements in psychiatric symptoms and global functioning, as well as cognitive gains (Allott et al., 2020; Vita et al., 2021). As evidence-based practices and treatments for addressing cognition proliferate, there is a growing need to understand how these can be effectively and efficiently implemented.

Implementation science is the rigorous study of how to support and improve the adoption, application and maintenance of evidence-based practices in routine care. It has been estimated that evidence-based practices take an average of 17 years to be integrated into routine health care (Morris et al., 2011). This research-to-practice gap has prompted an expansion of the field of implementation science, including the development of measures, frameworks and research designs to support high-quality implementation research. Implementation science represents an exciting avenue to improve the uptake of evidence-based practices and treatments for cognitive functioning in psychosis. This paper aims to provide an overview of key concepts and designs in implementation science and offer suggestions for the application of implementation science in the development and testing of cognition-focused treatments for people living with psychosis.

## Key concepts in implementation science

Implementation is a complex process, requiring consideration of contexts, including the individuals, teams, organizational settings and the systems in which practice is embedded (Helfrich et al., 2010). Implementation science seeks to capture information about the quality and efficiency of implementation in context, to build evidence about how to design and select implementation strategies that work. To this end, implementation science develops and rigorously tests implementation strategies, to gain knowledge about what implementation strategies work in certain contexts and why, as well as building generalizable knowledge that can be applied beyond the system under study, to help improve the implementation of evidence-based practices (Bauer et al., 2015). To date, implementation science has produced many ‘success stories’ across health and mental health sectors (Kilbourne et al., 2020), including for people affected by serious mental illness. For instance, the US-based Enhancing Quality-of-care In Psychosis (EQUIP) project has identified quality gaps in the treatment of psychosis and improved care by creating resources and education for clinicians, as well as champions, the addition of care managers and informatics systems (Brown et al., 2008). This research provides concrete examples of how implementation science can drive meaningful improvements in the availability of evidence-based interventions in mental healthcare.

To understand implementation research, it is useful to consider how it differs from conventional clinical research (see Table 1). Clinical research focuses on evaluating the effects (efficacy or effectiveness) of a treatment on patient outcomes, such as symptoms and functioning. By comparison, implementation research focuses on implementation of interventions and strategies to promote the uptake or adoption of evidence-based practices by providers or systems of care, including teams, units or services (Peters et al., 2013). Whereas clinical trials are typically designed to minimize the effect of confounding variables, implementation research considers the characteristics of the practice or treatment to be implemented, the people involved and the implementation context, all of which can play a part in implementation success or failure. Notably, implementation science focuses on improving care in ‘real-world’ settings, outside controlled clinical trial environments. This means that effectiveness is often investigated without excluding certain people or groups to control for causal effects. Compared to theory used in clinical research, which typically points to mechanisms of effectiveness within the patient, implementation models and frameworks tend to focus on system-level context, including cultural, economic, political, social, legal and physical environments, as well as organizational-level context that comprises a range of stakeholders and their relationships. All of these factors can influence implementation in complex health and mental health settings.

**Table 1.** Features of conventional clinical effectiveness research and implementation research.

Feature	Clinical effectiveness research	Implementation research
Aim	Understand processes and factors associated with health	Understand processes and factors associated with implementation
Framework, model, theory	Focused on mechanisms within the patient that mediate clinical outcomes; for example, biological, psychological	Focused on characteristics of the practice or treatment, people involved in implementation, process, context and interconnections that may influence implementation outcomes
Intervention	Evaluation of the clinical practice or treatment; highly controlled by strict protocol and safety guidance; for example, dose, delivery method	Evaluation of implementation intervention or strategy; for example, implementation strategy cost, dose, delivery method In addition, direct consideration is given to characteristics of the practice or treatment that may influence implementation outcomes; for example, adaptability, complexity, cost
Individuals	Focus on patients; typically controlled by strict inclusion/exclusion criteria; for example, medical history	Focus on the people involved in the implementation of the practice or treatment in ‘real world’ settings; for example, clinician knowledge, attitudes, identification with organization
Implementation context	Typically described as part of methodology in terms of clinical setting in which an evidence-based practice or treatment is delivered; for example, outpatient psychiatric unit in Melbourne, Australia	Often considered as a direct subject of investigation or in the design of implementation strategies being investigated; for example, inner context factors, such as organizational culture, as well as outer context factors, such as patient needs, health system design or external incentives
Implementation process	May or may not be described in full, typically in terms of what was done to prepare clinicians to deliver an evidence-based practice or treatment; for example, training	Described in full in terms of strategies used and steps taken during implementation. Includes consideration of activities leading up to and after implementation and the broader context and multiple groups of people involved; for example, planning, engaging, incentives, policy change, reminders
Outcomes	Unit of analysis: patient Focus on clinical and functional outcomes as indicators of effectiveness; for example, cognitive improvement, functioning	Unit of analysis: providers, teams/units, services or systems Focus on implementation outcomes, process and/or quality measures as indicators of implementation success; for example, acceptability, fidelity, adoption, sustainability

These differences are most obvious when considering clinical effectiveness and implementation research in their purest form. However, this does not mean that clinical and implementation researches are incompatible. At a minimum, implementation should be documented in detail across all research. There is overlap between process evaluation and implementation research, where both can seek to document the process of implementation and whether implementation has occurred as intended. Process evaluation is recognized as a component of implementation research, with both aiding in understanding how factors, such as intervention components, implementation strategies, resources and participants might impact outcomes (Limbani et al., 2019). As a field, implementation research extends on process evaluation, by seeking to unpack and understand the relative influence of different aspects of process and to test and refine processes and strategies to help improve implementation and clinical outcomes. There is increasing interest in blended approaches that combine implementation research alongside clinical effectiveness research, also known as hybrid designs (Curran et al., 2012) (see Table 2). Research using this approach has strong potential to accelerate the introduction of innovations into practice by producing more rapid translational outcomes, including insights into effective and pragmatic implementation strategies. Hybrid designs offer several options to help build the evidence for implementation alongside effectiveness, depending on a number of factors, such as: face validity, existing evidence and risks associated with the practice and implementation strategy, current implementation momentum and feasibility of implementation. Depending on the existing evidence-base and face validity, different hybrid designs may be suitable for a range of cognition-focused treatments for psychosis.

**Table 2.** Hybrid research designs offer a blended approach to clinical effectiveness and implementation research.

Feature	Non-hybrid clinical trial	Hybrid type 1	Hybrid type 2	Hybrid type 3	Non-hybrid implementation trial
Research aim/s	Determine the efficacy or effectiveness of a clinical practice/treatment compared to a control	Primary: determine the effectiveness of a clinical practice/treatment Secondary: understand the context for implementation	Co-primary aim: determine effectiveness of a clinical practice/treatment Co-primary aim: determine feasibility and potential utility of an implementation intervention/strategy	Primary aim: determine utility of an implementation intervention/strategy Secondary aim: assess clinical outcomes associated with implementation intervention/strategy	Determine effectiveness of an implementation intervention/strategy
When used	When a new clinical practice/treatment has been developed and tested for safety and effectiveness in pre-clinical trials or piloted with a small number of people If a practice or treatment is being delivered in a clinical setting, implementation factors could be assessed to inform future trials and/or implementation strategy development.	When there is • strong face validity for the clinical practice/treatment • a strong base of at least indirect evidence (e.g. data from different but associated populations) • minimal risk associated with the practice/treatment	When there is • reasonable expectations that the implementation intervention/strategy being tested is supportable in the context • reason to gather more data on the effectiveness of the clinical practice or treatment	When there is • evidence that the implementation intervention/strategy being tested is supportable in the context	When clinical effectiveness is established
			• strong face validity for the clinical practice/treatment and implementation intervention/strategy • a strong base of indirect evidence for the clinical practice/treatment and implementation intervention/strategy • minimal risk associated with the clinical practice/treatment and implementation intervention/strategy • ‘implementation momentum’ within the clinical system and/or the literature towards routine adoption of the clinical practice/treatment		
Example research questions	Primary question: What is the effect of cognitive remediation on cognitive, functional and quality of life outcomes for young people with first-episode psychosis?	Primary question: What is the effect of cognitive remediation on cognitive, functional and quality of life outcomes for young people with first-episode psychosis? Secondary question: What are the potential barriers and facilitators to implementing cognitive remediation in a youth mental health setting?	Co-primary question: What is the effect of cognitive remediation on cognitive, functional and quality of life outcomes for young people with first-episode psychosis? AND Co-primary question: Is training with supervision an acceptable strategy to support implementation of cognitive remediation in a youth mental health setting?	Primary question: What is the effect of implementation strategy (training with supervision versus training with supervision and champion intervention) on adoption of cognitive remediation by youth mental practitioners? Secondary question: Are clinical outcomes associated with each implementation strategy acceptable?	Primary question: Is fidelity to an implementation strategy (training with supervision and champion intervention) associated with improved adoption of cognitive remediation by youth mental practitioners?

Source: See Curran et al. (2012) for more information on hybrid designs.

As the field of implementation science continues to grow and evolve, rigorous implementation research frameworks, designs and measures are becoming available. The Expert Recommendations for Implementing Change study defined a comprehensive list of implementation strategies to enhance the adoption, implementation and sustainability of a clinical programme or practice, which can be used in implementation research and practice (Powell et al., 2015). Based on systematic reviews, the Implementation Outcome Repository (available at https://implementationoutcomerepository.org/) offers a useful collection of implementation outcomes rated for psychometric and methodological quality, as well as usability. A range of frameworks, theories and models are also available that support implementation research (Moullin et al., 2020; Nilsen, 2015). There is an effort to provide practical guidance on the application of implementation models and frameworks for data collection, analysis and research design (e.g. www.cfirguide.org, www.EPISframework.com). Most of these tools are freely accessible and, in many cases, can be used by researchers without specialist training in implementation science or practice. Implementation research is, however, inherently interdisciplinary, and clinical, public health and implementation researchers will benefit from working together.

## What is known about the implementation of treatments targeting cognitive functioning in psychosis?

Overall, research on the implementation of treatments that target cognitive impairment in psychosis is relatively limited (Bryce et al., 2021). There has been some research on the implementation of cognitive remediation for psychosis in adult mental health settings, which suggest that it is generally considered acceptable to clinicians (Medalia et al., 2019a, 2019b, 2021) and service users (Dark et al., 2018; Reeder et al., 2016). One study in a first-episode psychosis specialty care service found good adoption by staff and utilization by clients, with the programme reported as acceptable and feasible by clients (Lewandowski, 2021). This finding supports results of previous consumer-led research that showed cognitive remediation was acceptable and valued from the perspective of people with schizophrenia (Rose et al., 2008), and quality assurance data from US Psychiatric Centres, which showed a high level of satisfaction among people with serious mental illness receiving cognitive remediation as part of treatment (Medalia et al., 2019b). Another study of cognitive enhancement therapy for people with serious mental illness (88.3% schizophrenia-spectrum diagnosis) explicitly described implementation strategies, including external training and tele-observation (Faith et al., 2022). Authors of that study also reported on treatment adoption by patients, including measures of satisfaction and retention, but did not include any clinician-completed implementation measures, making it unclear how clinicians viewed the appropriateness of cognitive remediation or the implementation strategies used. Where studies have considered clinician perspectives, results have shown that clinicians find cognitive remediation to be acceptable (Medalia et al., 2021). Other research suggests, however, that clinician knowledge and/or attitudes could be a barrier to uptake. One German study with mental healthcare professionals (*N* = 657) investigating the implementation of current evidence- and consensus-based guidelines for schizophrenia found that cognitive remediation was perceived as effective by less than one-half and referred by less than one-fifth of respondents, deviating from a strong guideline recommendation (Gaigl et al., 2021). This is consistent with the results of an implementation case study of cognitive remediation in Icelandic early psychosis services, where clinical directors reported staff attitudes as one of the most important factors influencing implementation, along with patient needs (Vidarstottir et al., 2021). Overall, available findings suggest that implementation strategies for behavioural cognitive remediation treatments may benefit from focusing on supporting engagement from both clinicians and patients. An implementation project in the US integrating cognitive remediation in adult Psychiatric Centres conducted facility needs assessments to understand potential demand and available infrastructure, staff training, an educational campaign aimed at building a culture of cognitive health in services, operationalization of referral and screening processes and fidelity monitoring (Medalia et al., 2019b). Cognitive remediation was successfully implemented at 13 of 16 adult Psychiatric Centres in rural and urban settings across New York state, with quality assurance data indicating that the treatment was acceptable, sustainable and effective. In Iceland, research on cognitive remediation delivered in early psychosis services included an effectiveness study and an implementation case study, which found cognitive remediation was successfully integrated into routine care using a range of strategies including training and education, supported by academic partnerships and a local implementation team (Vidarstottir et al., 2021). However, without testing different implementation strategies and comparing implementation outcomes, it is not currently clear which strategy is most appropriate in which context.

The relatively limited research on the implementation of cognitive remediation is problematic given the evidence for its clinical effectiveness (Bryce et al., 2021; Vita et al., 2021). Encouragingly, a multi-centre randomized controlled trial is underway in the United Kingdom, testing three different methods of providing cognitive remediation in early intervention psychosis services (Wykes et al., 2018). Interviews with providers to understand barriers and facilitators to the introduction of cognitive remediation have identified resource shortages as the main barrier for implementation, with the evidence-base for cognitive remediation and the recognition of clinical need seen as the main facilitator (Lammas et al., 2022). This is an example of a hybrid trial with a primary focus on patient outcomes (e.g. cognition, functioning) and a secondary process evaluation to understand the context of implementation and the satisfaction of patients and clinicians involved in implementation.

Previous research in the field of psychosis provides some insight into factors that impact the implementation of behavioural treatments. Several systematic and narrative reviews have summarized barriers and enablers to implementation of family involvement in treatment for psychosis. They point to the importance of organizational culture, seeing family as an important part of care and highlight the need for cultural adaptation of family-focused care (Asmal et al., 2011; Bucci et al., 2016; Eassom et al., 2014; Ince et al., 2016; Selick et al., 2017; Sin and Norman, 2013). Though research on barriers and facilitators to implementing guidelines for the treatment of schizophrenia spectrum disorders is scarce, available literature suggests the existence of barriers at the level of the organization, such as insufficient support from managers (Berry and Haddock, 2008). Other barriers to implementation of psychological treatments for schizophrenia may be lack of clinician skills and client reluctance to engage with services (Berry and Haddock, 2008). Similarly, research on barriers to implementation of cognitive behavioural therapy (CBT) for psychosis has found that organizational barriers are most frequently reported, followed by barriers met by staff and service users (Ince et al., 2016). Altogether, available research suggests a need for further knowledge of the potential and actual implementation barriers at organizational and individual levels, with a view to designing strategies that will overcome these.

## Future directions

Given that clinical guidelines make specific recommendations for addressing cognition in psychosis (Galletly et al., 2016; Norman et al., 2017), more research is warranted to understand and address gaps between current practice and best practice. Focused implementation research is needed to understand the most appropriate implementation strategies to support adoption and sustainability in routine care, particularly for the clinical guidelines and treatments with established efficacy in psychotic disorders, such as cognitive remediation and compensation. Where there is still a need to build the evidence for clinical effectiveness, researchers can consider implementation–effectiveness hybrid designs (Curran et al., 2012). For pure clinical trials being delivered in a clinical setting, implementation factors can be assessed to inform future trials and implementation strategy development.

An area that implementation science may be of particular value is in understanding and addressing patient factors that can influence engagement with treatment. Early and sustained engagement in treatment can improve outcomes for people living with serious mental illness, including psychosis, with benefits for symptoms, relapse and functioning (Dixon et al., 2016). Service disengagement for people with psychosis is prevalent, with estimates from 12% up to 53% from across early psychosis and other settings that treat psychosis (Doyle et al., 2014; Kreyenbuhl et al., 2009; Mascayano et al., 2020). Patient motivation is seen as key contributor, with impairments in motivation a common feature of psychotic disorders (Brett et al., 2018). Research on cognitive remediation and compensation for people with psychosis also shows relatively high levels of dropout (Allott et al., 2020; Wykes et al., 2011), with some evidence that participants with schizophrenia ‘felt worse’ if they did not perceive improvement through cognitive remediation (Rose et al., 2008) and that computer exercises could be ‘repetitive’ and ‘boring’ (Lewandowski, 2021). Overall, however, the available evidence suggests that people with psychosis find cognitive remediation acceptable (Lewandowski, 2021; Rose et al., 2008). In fact, treatment engagement is complex phenomena, involving interaction between numerous factors, such as attitude, perceived utility of treatment and practical influences, such as service accessibility. (Dixon et al., 2016). Implementation science can help to build understanding of the relative influence of these different factors, with leading frameworks including patient characteristics (e.g. motivation) as well as appropriateness of the treatment for specific patient groups (Aarons et al., 2011; Damschroder et al., 2022). Further research to understand the relationships between these factors, implementation outcomes and patient outcomes, can contribute to the design of evidence-based strategies to improve adoption and engagement.

Future research on the implementation of evidence-based practices and treatments targeting cognition in psychosis should seek to engage a range of stakeholders who will influence and/or be influenced by implementation. This might include decision-makers, such as service leaders, commissioners or policy-makers, clinicians, allied healthcare professional, peer support workers and people living with psychosis and their support networks. By working together, clinical researchers and implementation scientists, along with other stakeholders, can bring together the expertise to build the implementation evidence that can help improve outcomes for people with psychosis.
